# Cutaneous Alternariosis in Immunosuppressed Patients Treated with Photodynamic Therapy and Oral Antifungals, a Synergistic Strategy

**DOI:** 10.3390/ph17020245

**Published:** 2024-02-13

**Authors:** Pedro Gil-Pallares, Tamara Gracia-Cazaña, Marcial Álvarez-Salafranca, Marcos Antonio Gorgojo, Mar García-García, Antonio Beltrán-Rosel, Manuel Almenara-Blasco, Alba Navarro-Bielsa, Yolanda Gilaberte

**Affiliations:** 1 Department of Dermatology, Miguel Servet University Hospital, 50009 Zaragoza, Spainmalvarezs@salud.aragon.es (M.Á.-S.); malmenara@salud.aragon.es (M.A.-B.); ygilaberte@salud.aragon.es (Y.G.); 2 Department of Surgery and Medical-Surgical Specialities, Universidad de Santiago de Compostela, 15705 Santiago de Compostela, Spain; 3 Instituto de Investigación Sanitaria Aragón (IIS Aragón), 50009 Zaragoza, Spain; 4 Department of Dermatology, San Jorge Hospital, 22004 Huesca, Spain; 5 Department of Pathology, Hospital Clínico Universitario Lozano Blesa, 50009 Zaragoza, Spain; mgarciagarc@salud.aragon.es; 6 Department of Microbiology, Hospital Clínico Universitario Lozano Blesa, 50009 Zaragoza, Spain; 7 Department of Dermatology, University of Zaragoza, 50009 Zaragoza, Spain

**Keywords:** *Alternaria*, photodynamic therapy, PDT, cutaneous alternariosis, 5-aminolevulinic acid, methyl-aminolevulinate, methylene blue

## Abstract

Cutaneous alternariosis is a rare condition, more frequently presented in immunocompromised patients, which usually requires long courses of systemic antifungals that may interact with other medications. The presented series shows three cases of cutaneous alternariosis in immunocompromised patients and organ transplant recipients that were successfully treated with photodynamic therapy and oral antifungals, allowing a reduction in the systemic treatment duration and therefore decreasing the risk of side effects and drug interactions.

## 1. Introduction

*Alternaria* is a dematiaceous fungus, with widespread presence in the natural environment, that causes phaeohyphomycosis, a relatively infrequent human infection. This melanized mold can lead to a diverse range of clinical conditions, varying from localized manifestations in specific organs to more widespread systemic infections [1]. *Alternaria* infects mainly immunocompromised hosts; although there have been occasional instances of infections in individuals with healthy immune systems [2,3], these cases seldom progress to invasive diseases [4].

The skin and the subcutaneous tissues are the most frequently infected sites by this fungus, although other types of infections such as oculomycosis, sinus infections, onychomycosis, and invasive diseases have been described [1].

The treatment of cutaneous alternariosis includes, when feasible, the reduction of immunosuppression, in addition to long courses of antifungals, which often interact with the immunosuppressants taken by organ transplant recipients (OTRs). Excision of the lesions can also be an option in certain cases, often combined with systemic treatment [5].

Recently, some authors have shown the use of antimicrobial photodynamic therapy (PDT) as an effective treatment of phaeohyphomycosis [6,7], and also chromoblastomycosis [4,8]. PDT is based on the use of photosensitizing molecules that end up generating reactive oxygen species (ROS) that destroy the target cells when being irradiated with light of a suitable wavelength and a proper dose [9]. Some reports have shown that PDT is effective in the treatment of cutaneous infections due to pathogens such as multi-resistant mycobacteria, in combination with systemic treatments [10]. Moreover, it also offers the possibility of a reduction in the duration of systemic treatment of fungal infections such as onychomycosis [11]. In relation with cutaneous alternariosis, Liu et al. [4] reported a case of an infection due to *Alternaria alternata* treated with oral itraconazole followed by PDT with complete healing. Moreover, the combination of systemic antifungal and PDT has also been described in other cutaneous fungal infections with promising results [6,7,8].

We present three additional cases of cutaneous alternariosis in immunocompromised patients, two of them OTRs, treated with systemic antifungals and PDT (Table 1), and a review of the literature about the role of PDT in the treatment of phaeohyphomycosis and chromoblastomycosis. In our series, the combination of both treatment modalities allowed shortening the antifungal treatment duration, diminishing the possibility of interactions with other drugs, especially in the ORTs, and side effects.

## 2. Case 1

**Figure 1:**
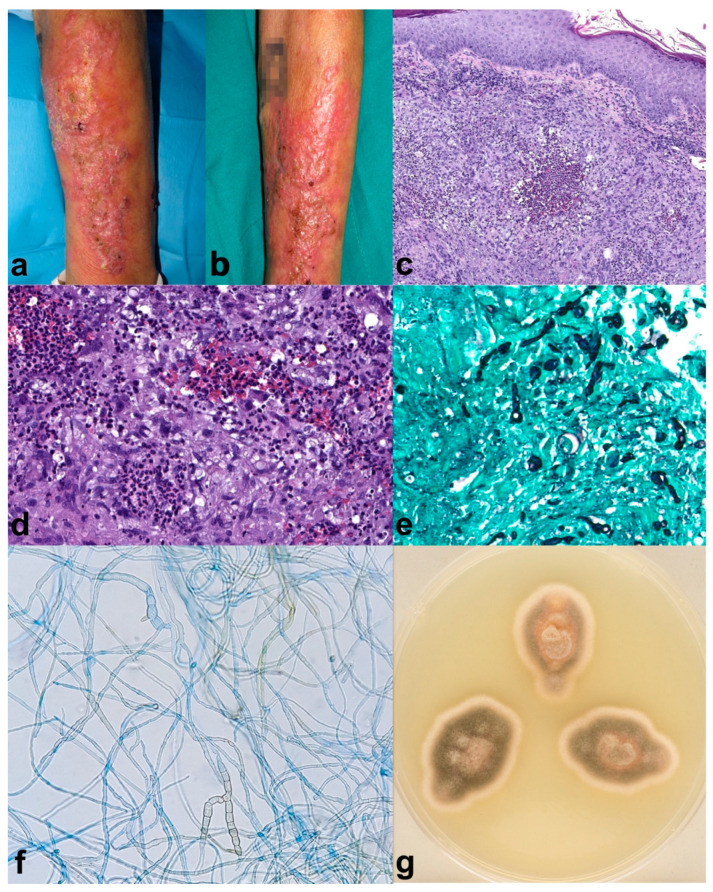
Case 1. (**a**,**b**) Erythematous verrucous plaques on the left forearm. (**c**) (H-E 8×) Histopathological analysis showed light acanthosis, with mild hyperkeratosis and foci of parakeratosis; at the dermal layer, there was granulomatous perifollicular inflammation with multinuclear, surrounding broken follicles. (**d**) (H-E 20×) Some yeast forms can also be identified. Grocott (**e**) (20×) and PAS staining revealed structures compatible with hyphae, and Ziehl–Nielsen was negative. (**g**) Macroscopic appearance of the culture showed a grey-olive green color and woolly texture colonies. (**f**) (40×) The septate, brown hyphae seen microscopically were compatible with *Alternaria* spp. *Alternaria infectoriae* was identified by PCR sequencing. Partial improvement was seen five months after Itraconazole (100 mg/12 h for 1 month and Itraconazole 50 mg/12 h for 4 months), which was followed by two sessions of 5-aminolevulinic acid (5-ALA) daylight photodynamic therapy, achieving resolution of the lesions (no photography available). The patient died shortly after due to COPD worsening.

A 70-year-old male with a personal history of Chronic Obstructive Pulmonary Disease (COPD), requiring frequent courses of oral steroids (30–40 mg of prednisone for 10 to 14 days every month), diabetes mellitus type 2 with poor control, and larynx squamous cell carcinoma. The patient developed verrucous plaques on the left forearm for four months before consulting (Figure 1a,b). Moreover, he mentioned gardening often and outdoor farming activities. The histopathological analysis showed light acanthosis, with mild hyperkeratosis and foci of parakeratosis; at the dermal layer, there was granulomatous perifollicular inflammation with multinuclear, surrounding broken follicles (Figure 1c,d). Grocott (Figure 1e) and PAS staining revealed structures compatible with hyphae, and Ziehl–Nielsen was negative. Tissue culture showed a grey-olive green color and woolly texture colonies (Figure 1g). Microscopic study showed septate, brown hyphae compatible with those of *Alternaria* spp. (Figure 1f). *Alternaria infectoriae* was identified by PCR sequencing. The patient received one month of itraconazole 100 mg twice daily, and fivefour months of 50 mg twice daily achieving partial improvement, followed by two sessions of daylight photodynamic therapy (DL-PDT) with 5-aminolevulinic acid (5-ALA, Ameluz^®^) consistent of 30 min of incubation followed by 2 hours of exposure to sunlight, once every 10 days. Complete healing was achieved after de DL-PDT, with no remission relapse after two months. The patient died shortly after due to COPD worsening.

## 3. Case 2

**Figure 2:**
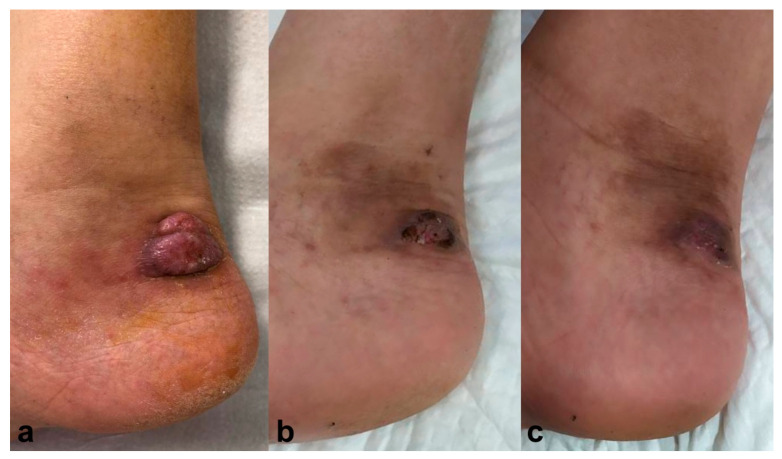
Case 2. (**a**) Erythematous-violaceus tumoral lesion on the lateral side of the right ankle. (**b**) Resolution six months after simultaneous treatment with voriconazole 400 mg/day for three months and photodynamic therapy with methyl-aminolevulinate (Metvix^®^). Resolution after six months and no recurrence after twelve months follow-up (**c**).

A 62-year-old male, treated with tacrolimus (0.1 mg/kg/day) and 10 mg/day of prednisone due to a kidney transplant, presented with a tumoral lesion on the lateral side of his right ankle two months ago (Figure 2a). Histopathology analysis showed hyphae compatible with phaeohyphomycotic infection, and culture confirmed *Alternaria* spp. Simultaneous treatment with voriconazole 400 mg/day for three months and PDT with methyl-aminolevulinate (MAL-PDT), Metvix^®^, was started with 1 hour incubation, progressively increasing the time to 3 hours; and subsequent illumination with Aktilite^®^ increasing fluences from 37 to 74 J/cm^2^. MAL-PDT was performed once a week, with a total of 12 sessions, achieving complete resolution after six months, with no relapse after twelve months follow-up (Figure 2b).

## 4. Case 3

**Figure 3:**
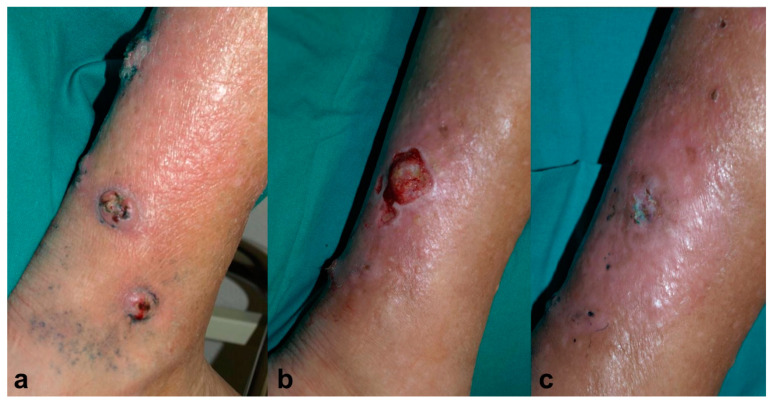
Case 3. (**a**) Ulcerated lesions on the right leg surrounded by grey-blue macules. (**b**) Partial resolution after 11 sessions of photodynamic therapy with methyl-aminolevulinate (Metvix^®^) (MAL-PDT) and 10 sessions of photodynamic therapy with methylene blue (MB-PDT). (**c**) Complete healing after 1.5 months of simultaneous treatment with voriconazole 400 mg/day and MB-PDT (6 sessions), leaving mild erythema and residual hyperpigmentation.

An 81-year-old male, recipient of a kidney transplant, under treatment with tacrolimus (0.2 mg/kg/day) and 5 mg/day of prednisone, was referred to the dermatology clinic for four ulcerated lesions surrounded by grey-blue macules on his right leg for the past month (Figure 3a). A skin biopsy showed findings consistent with deep fungal infection and *Alternaria* spp. grew in the tissue culture. It was decided to treat the patient with MAL-PDT, Metvix^®^, with three hours of incubation followed by illumination with Aktilite^®^ 37 J/cm^2^ (twice weekly). After 11 sessions, he showed partial improvement, but the culture was still positive; therefore, the photosensitizer was switched to methylene blue 1% solution (MB-PDT) with 30 minutes of incubation under occlusion and illuminated with Aktilite^®^ (fluence 74 J/cm^2^) once a week, and exposure to daylight for half an hour every day until the disappearance of the blue color. After 10 sessions of MB-PDT, since the biggest lesion persisted, voriconazole 400mg per day was added to MB-PDT (Figure 3b). After 1.5 months, the lesion was clinical and microbiologically cured, and voriconazole also had to be discontinued due to acute renal failure. No recurrence had been presented after three years of follow-up (Figure 3c).

## 5. Discussion

Cutaneous alternariosis is caused by the dematiaceous fungus *Alternaria*, which is frequently isolated in opportunistic deep cutaneous mycoses [5]. *Alternaria* is widely distributed and can be found in plants, water, soil water, or objects, among others [2]. The most frequent transmission mechanism in cutaneous infections is direct inoculation during or after a traumatism, commonly causing phaeohyphomycosis. However, cases of chromoblastomycosis due to *Alternaria*, characterized by the histopathologic finding of sclerotic or muriform cells, have also been reported [4,12,13]. The clinical presentation is usually non-specific and asymptomatic and may present as small papules or nodules, multinodular plaques, ulcers, cellulitis, or even blisters, usually in exposed areas of outdoor workers, as they are more prone to trauma [5,6].

Histopathological study often shows a dense dermal suppurative granulomatous infiltrate and fungal structures, which are more evident with different stains such as Grocott, Periodic acid–Schiff (PAS), or Masson-Fontana. These findings are enough to guide the management, which commonly involves empirical treatment with high doses of antifungals. Confirmation of the fungal species requires culture or direct sequencing [5].

Surgery can be an effective treatment option; however, it might not be enough or possible in some cases such as in large and multifocal cases that require a combination with systemic antifungals [5], or certain locations in which the surgery would be too aggressive, or in which healing would be difficult.

High doses of antifungals for several months are recommended to treat cutaneous alternariosis. Itraconazole 400 mg/day or voriconazole 400 mg/day are two of the most common options [5]. Nevertheless, the use of these treatments in OTRs requires close monitoring due to their interaction with calcineurin inhibitors, as well as other adverse reactions such as hepatotoxicity. A combination of surgery plus different antifungals is commonly needed to achieve cure rates around 90% due to adverse events to the antifungal drugs or lack of response to them [5], which is emerging as a concern even in immunocompetent patients [2].

PDT has been shown to be effective in different types of infections including bacterial infections [9], fungal infections [11,14], multi-resistant mycobacteria, or other pathogens [10,15]. The antimicrobial efficacy of PDT has been extensively explored through numerous in vitro studies since the 1990s, and unlike traditional antibiotics, PDT’s diverse cellular targets make resistance development less likely [16].

Recently, there have been reports of encouraging outcomes achieved through the utilization of PDT (using methylene blue as a photosensitizer rather than ALA) in the management of chromoblastomycosis [17,18]. Moreover, several authors have also shown the utility of PDT with ALA as an adjuvant of antifungal therapy for the treatment of different phaeohyphomycosis and chromoblastomycosis (Table 2).

Yang and colleagues [19] documented a challenging case of chromoblastomycosis caused by *F. monophora* that showed improvement following treatment with ALA-PDT. Moreover, Liu et al. [4] described a 50-year-old man with a chromoblastomycosis because of *Alternaria alternata*, successfully treated with a short course of systemic itraconazole and subsequent 5-aminolevulinic acid-photodynamic therapy.

The presented case series shows similar results of PDT in phaeohyphomycosis due to *Alternaria* and highlights the challenges of diagnosing and managing opportunistic co-infection in an immunocompromised host. As previously described in fungal infections such as onychomycosis [11], PDT with MAL or 5-ALA potentiates the effects of the systemic antifungals, both sequentially or simultaneously, permitting a reduction in the duration of the systemic treatment, and therefore, diminishing the possibility of drug adverse events and interactions. In addition, the beneficial effects of PDT on host tissues, such as growth factor stimulation and immune response enhancement, potentially foster improved wound healing, particularly in common presentations of this condition such as chronic ulcers [20].

**Table 2:** Summary of previous reports of chromoblastomycosis and phaeohyphomycosis treated with PDT. CBM: chromoblastomycosis. PHM: Phaeohyphomycosis. ALA: 5-aminolevulinic acid. N/S: not specified. The sign “+” indicates simultaneously.

Study	Pathogen	Oral Treatment	PDT Photosensitizer (Incubation)	Source of Light	Total Number of Sessions	Outcome
Wang et al. 2023 [21]	*C. lunata* (PHM)	None (surgery + PDT)	20% ALA (4 h)	LED 635 nm (80 J/cm^2^)	3 (every 9 days)	Healing
Yang et al. 2020 [22]	*F. monophora* (CBM)	Itraconazole 400 mg/d (2 months) + PDT	20% ALA (2 h)	LED 630 nm (90 J/cm^2^)	3 (every 10 days)	Healing
Lan et al. 2021 [23]	*F. monophora* (CBM)	Terbinafine 250 mg/d, Itraconazole 400 mg/d (4 months) and isotretinoin 20 mg/d (1 month), before ALA-PDT	20% ALA (3 h) + previous vaporization of hyperkeratosis with CO_2_ laser	LED 633 nm (96 J/cm^2^)	4 (weekly)	Improvement
Liu et al. 2019 [7]	*E. spinifera* (PHM)	Itraconazole 200 mg/d + terbinafine 250 mg/d (5 months) + PDT	20% ALA (4 h)	LED 633 nm (120 mW/cm^2^, 25 min)	3 (weekly)	Healing
Hu et al. 2019 [8]	*F. nubica*	Itraconazole 200 mg/d (1 year) + PDT	20% ALA (4 h)	LED 635 nm (36.8 mW/cm^2^)	4 (weekly)	Improvement
Hu et al. 2019 [8]	*F*. *pedrosoi*	Terbinafine 250 mg/d (8 months) + PDT	20% ALA (4 h)	LED 635 nm (36.8 mW/cm^2^)	4 (weekly)	Healing
Hu et al. 2019 [8]	*F*. *pedrosoi*	Itraconazole 200 mg/d + terbinafine 250 mg/d (2 years) + PDT	20% ALA (4 h)	LED 635 nm (36.8 mW/cm^2^)	3 (weekly or every 2 weeks)	Improvement
Hu et al. 2019 [8]	*F*. *monophora*	Terbinafine 250 mg/d	20% ALA (4 h)	LED 635 nm (36.8 mW/cm^2^)	18 (weekly)	Healing
Hu et al. 2019 [8]	*F*. *monophora*	Terbinafine 250 mg/d	20% ALA (4 h)	LED 635 nm (36.8 mW/cm^2^)	10 (weekly)	Improvement
Huang et al. 2019 [24]	*F. pedrosoi* (CBM)	Itraconazole 400 mg/d (2 moths) before PDT	10% ALA (4 h)	LED 633 nm (80–100 mW/cm^2^, 25 min)	6 (every 2 weeks)	Healing
Liu et al. 2014 [4]	*Alternaria alternata* (CBM)	Itraconazole 400 mg/d (15 weeks) before PDT	20% ALA (3 h)	LED 633 nm (80 mW/cm^2^)	3	Healing
Yang et al. 2012 [19]	*F. monophora* (CBM)	Terbinafine 250 mg/d (5 months) and voriconazole 200 mg/d (2 months) + PDT	ALA	N/S	10 (weekly)	Improvement
Pereira Lyon et al. 2011 [17]	10 cases of CBM	Itraconazole after PDT	20% methylene blue preparation in Eucerin cream (4 h)	LED 660 nm (28 J/cm^2^)	6 (weekly)	Improvement with PDT and healing after itraconazole

## 6. Conclusions

PDT has been shown to be effective for the treatment of various types of infections including cutaneous fungal infections. The presented case series, as well as previous reports, supports its role as a simple and effective treatment for cutaneous alternariosis and other cutaneous fungal infections, which has a synergistic effect with oral antifungals. Combination of PDT with systemic antifungals allows an important reduction in the treatment duration and the healing time. By shortening the systemic antifungal treatments, the possible interactions with other drugs are also reduced, increasing the safety of the treatment. This is particularly important in certain populations, such as organ transplant recipients, who need immunosuppressive treatments that require monitoring when taking certain systemic antifungals and are more susceptible to these types of opportunistic infections.

## Figures and Tables

**Table 1:** Summary of the cases of cutaneous *Alternaria* infection treated with photodynamic therapy and systemic antifungals. COPD: Chronic Obstructive Pulmonary Disease. PDT: photodynamic therapy. 5-ALA: 5-aminolevulinic acid (Ameluz^®^). MAL: methyl-aminolevulinate (Metvix^®^).

n	Sex/Age	Immunosuppression	Identified Pathogen	Oral Antifungal	PDT Type (Photosensitizer)	Timing of PDT	Outcome	Follow-Up
1	♂ 70	COPD (oral steroids), larynx SCC	*Alternaria infectoriae*	Itraconazole 100 mg/12 h for 1 month Itraconazole 50 mg/12 h for 4 months	Daylight PDT (5-ALA) 2 sessions ^1^	Following oral antifungal	Partial healing after itraconazole and complete resolution after daylight PDT	2 months (death due to COPD)
2	♂ 62	Kidney transplant (tacrolimus)	*Alternaria* spp.	Voriconazole 400 mg/day 3 months	Conventional PDT (MAL) 12 sessions ^2^	Simultaneously with oral treatment	Resolution after 6 months	12 months (no relapse)
3	♂ 81	Kidney transplant (tacrolimus, oral steroids)	*Alternaria* spp.	Voriconazole 400 mg/day 1.5 months	Conventional PDT (MAL) 11 sessions ^3^, (Methylene blue) 16 sessions ^4^	Simultaneous oral antifungal and PDT from the 10th Methylene blue session	Partial resolution after PDT, and complete healing after 1.5 months from the combination with oral antifungal	3 years (no relapse)

^1^ Thirty minutes of incubation followed by two hours of exposure to sunlight, once every 10 days. ^2^ Incubation time starting at 1 hour and progressively increasing to 3 hours, and subsequent illumination with Aktilite^®^ CL 128 increasing fluence from 37 to 74 J/cm^2^, once a week. ^3^ Three hours of incubation followed by illumination with Aktilite^®^ CL 128 (fluence 37 J/cm^2^), twice weekly. ^4^ Thirty minutes of incubation and illumination with Aktilite^®^ CL 128 (fluence 74 J/cm^2^) once a week, and exposure to daylight half an hour every day until fading of the blue color.

## Data Availability

Data is contained within the article.
